# Safety and Feasibility Colorectal Anastomosis Protocol Implementation: Results from the CASPI Single-Arm Pilot Study

**DOI:** 10.3390/cancers18030400

**Published:** 2026-01-27

**Authors:** Ernesto Barzola, Lidia Cornejo, Judith Luquín, David Julià, Núria Gómez, Anna Pigem, Olga Delisau, Eloi Maldonado, Ramon Farrés, Pere Planellas

**Affiliations:** 1 Colorectal Surgery Unit, Department of General and Digestive Surgery, University Hospital of Girona, 17007 Girona, Spain; 2 Hospital Clinic Barcelona, Faculty of Medicine, University of Barcelona, 08036 Barcelona, Spain; 3 General and Digestive Surgery Research Group, Girona Biomedical Research Institute (IDIBGI), 17190 Girona, Spain; 4 Department of Medical Sciences, Faculty of Medicine, University of Girona, 17071 Girona, Spain

**Keywords:** colorectal cancer, anastomotic leak, intraoperative endoscopy, indocyanine green

## Abstract

Background/Objectives: Anastomotic leakage (AL) is a major complication of colorectal surgery. Despite multiple identified risk factors, no single strategy has proven fully effective in preventing AL. This single-arm pilot study aims to evaluate the feasibility, safety, and adherence of a multimodal colorectal anastomosis assessment protocol (CASPI) in patients undergoing surgery for colorectal cancer. Methods: This prospective descriptive interventional single-arm pilot study included patients diagnosed with colorectal cancer who underwent surgical resection. The CASPI protocol consists of five steps: (1) indocyanine green (ICG) perfusion assessment, (2) doughnut integrity checking, (3) air leak testing, (4) intraoperative flexible endoscopy, and (5) postoperative flexible sigmoidoscopy. Results: A total of 34 patients were included. The median age was 63.5 years, and the median BMI was 27.7 kg/m^2^. Twenty-seven patients had rectal tumors, and 66.7% received neoadjuvant therapy. Adherence to the protocol was 100% intraoperatively and 88.2% postoperatively. Adequate perfusion by ICG was confirmed in 94.1% of cases; intact anastomotic doughnuts were obtained in all procedures. Intraoperative endoscopy showed Grade 1 mucosa in 76.5% of patients and Grade 2 in 23.5%. No complications related to the CASPI protocol were observed. Stoma closure was performed in all patients with temporary ileostomy. Conclusions: Implementation of the CASPI protocol in colorectal surgery demonstrated excellent feasibility, high adherence, and strong safety. These findings support its further evaluation in larger, controlled studies designed to assess clinical effectiveness in the incidence of anastomotic complications.

## Simple Summary

Anastomotic leakage is one of the most serious complications after colorectal surgery and can greatly affect patients’ recovery and quality of life. Our team developed the CASPI protocol, a step-by-step method that allows surgeons to carefully assess the blood supply, integrity, and safety of the bowel connection during and after the operation. In this pilot study, we applied the protocol to patients with colorectal cancer to evaluate its safety, ease of use, and consistent applicability. The CASPI protocol demonstrated high adherence without causing any complications. These results suggest that the CASPI protocol is safe and support its feasibility for routine clinical practice.

## 1. Introduction

Anastomotic leak (AL) remains one of the most serious complications after colorectal surgery, with reported rates ranging from 5% to 20% [1,2,3,4,5]. Multiple factors have been associated with its occurrence, including patient characteristics, tumor location, surgical instruments, surgeon experience, and the microbiota [2,3,4,5]. Preventive measures such as tension-free anastomosis, ensuring adequate blood supply, and systematically assessing potential technical failures are essential to reduce AL rates and related complications [4,5].

Several intraoperative methods have been incorporated into colorectal cancer surgery to reduce the risk of AL, including the air leak test (ALT), indocyanine green (ICG) fluorescence imaging, and intraoperative endoscopy [6,7,8,9,10]. ALT detects only mechanical defects, and current evidence regarding its effectiveness remains limited due to heterogeneous study designs [11,12,13].

ICG enables real-time angiography, helping surgeons identify well-perfused segments before transection [9,10]. Sufficient evidence now suggests that ICG fluorescence imaging reduces AL rates, particularly in left-sided and rectal resections [13,14,15].

Intraoperative endoscopy is another tool that allows direct evaluation of anastomotic integrity and the detection of mechanical defects. A meta-analysis by Aly et al. [16] showed that intraoperative functional endoscopy reduces postoperative complications from 6.9% to 3.5% compared to other conventional anastomosis checking methods. Farzaneh et al. [17] suggested that its systematic implementation can lead to a reduction in anastomotic leaks. However, further evidence is still needed to fully establish the value of intraoperative endoscopy, especially in high-risk patients [16,18,19].

Postoperative sigmoidoscopy enables early identification of complications at the colorectal anastomosis. It provides direct visualization of tissue integrity, allowing timely interventions when necessary. Systematic use of this approach may improve patient outcomes and reduce the severity of anastomotic issues [20]. However, there is still no consensus regarding its routine implementation.

To date, each verification method has been assessed individually. Ideally, an optimal evaluation of anastomotic integrity should combine the assessment of both mechanical integrity and perfusion. To integrate all these methods, we developed the CASPI protocol, which assesses anastomoses through a combination of perfusion imaging, mechanical testing, and intraoperative and postoperative endoscopic evaluation.

This pilot study aimed to evaluate the safety and feasibility of implementing the Colorectal Anastomosis Safety Protocol (CASPI) in the clinical practice of a colorectal surgery unit.

## 2. Materials and Methods

### 2.1. Trial Design

This prospective, observational, descriptive, interventional, single-arm, pilot study was conducted in the specialized Colorectal Surgery Department at Dr. Josep Trueta University Hospital of Girona from June 2022 to February 2023.

### 2.2. Study Population

Eligible participants were adults aged ≥18 years diagnosed with adenocarcinoma or adenoma who underwent elective surgery with the intention of R0 resection and anastomosis formation. Exclusion criteria included inability to provide informed consent, planned formation of a terminal ostomy, and loss to follow-up.

### 2.3. Procedure

Patients with locally advanced rectal tumors (cT3/cT4 and/or cN1/cN2) received total neoadjuvant theraphy or long-course preoperative radiotherapy, delivered in daily fractions of 1.8 Gy for a total dose of 45–50.4 Gy, combined with chemotherapy. One patient with synchronous metastatic disease was treated with preoperative chemotherapy alone.

All surgeries were performed using minimally invasive approaches, including laparoscopy, robotic surgery, and transanal total mesorectal excision (TaTME), by surgeons experienced in minimally invasive colorectal surgery and adhering to oncological surgical principles. The circular stapler used was Ethicon^TM^ Circular Stapler CDH (Ethicon, Somerville, NJ, USA). Temporary stoma formation was based on the surgeon’s criteria.

### 2.4. CASPI Protocol

**Figure 1:**
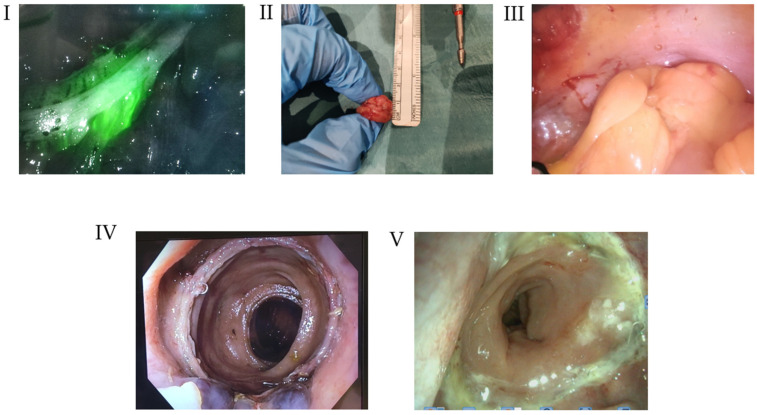
CASPI protocol steps. (I) Assessment of perfusion using ICG angiography; (II) Anastomosis Integrity Assessment—Doughnuts integrity; (III) Tightness Test—Air leak test: (IV) Intraoperative Endoscopic Evaluation; and (V) Postoperative endoscopy evaluation.

Following the principles of oncological surgery, the methods included in the CASPI protocol are described below (Figure 1).

(1) Perfusion Assessment—Indocyanine Green (ICG)

The proximal colon was inspected under conventional white light, and the anticipated point of perfusion change was marked. ICG was then administered intravenously through a peripheral line at a dose of 0.2 mg/kg (5 mg/mL) diluted in 10 mL of sterile water. After a 30 s interval, fluorescence angiography was performed using the Olympus system or Firefly technology (Da Vinci System) to assess colonic perfusion. Colonic transection was carried out precisely at the identified point of perfusion change.

(2) Anastomotic Integrity Assessment—Doughnut Integrity (DI)

After anastomosis creation, both the proximal and distal doughnuts were carefully examined. The width and height of each segment were measured, and photographic documentation was obtained.

(3) Tightness Assessment—Air Leak Test (ALT)

An initial air leak test was performed using a 50 mL wide-cone syringe to evaluate anastomotic tightness. Ambient air (50 mL) was aspirated and injected through a rectal tube while the anastomosis was inspected laparoscopically. The procedure was repeated with an additional 50 mL of air to confirm integrity.

(4) Intraoperative Endoscopy (IOE)

Anastomotic evaluation was performed using flexible endoscopy (colonoscope) by a colorectal surgeon trained in flexible endoscopy. The assessment included mucosal color, degree of edema, and degree of ischemia, as previously described by Sujatha-Bhaskar [21].

A second transabdominal pneumatic leak test was conducted under full endoscopic distension to confirm staple line integrity. Based on endoscopic findings or air leak testing results, intraoperative modifications to the anastomosis were performed at the surgeon’s discretion. This step enables the surgical team to identify any minor mucosal irregularities, bleeding points, or tension-related issues that might not be evident from perfusion or mechanical testing alone.

(5) Postoperative Endoscopy (PE)

Postoperative evaluation of the anastomosis was performed using a flexible endoscopy (sigmoidoscope) (Karl Storz–Endoskope, Tuttlingen, Germany) 15–21 days after surgery, without sedation. This step allows the identification of any anastomotic defects or late anastomotic leakage and provides an objective assessment of anastomotic healing.

### 2.5. Outcomes

The primary outcome was to assess the adherence of implementing the CASPI protocol in a colorectal unit by measuring adherence rates.

The secondary outcome was to evaluate the feasibility and safety of the CASPI protocol by monitoring adverse events during intraoperative and postoperative assessment of colorectal anastomoses.

### 2.6. Sample Calculation

As this was a single-arm pilot and exploratory study, no formal sample size calcula-tion was performed. According to the recommendations of Kieser and Wassmer [22], who suggested enrolling 30–40 patients for two-group pilot studies, we aimed to include at least 30 patients in the present study.

### 2.7. Demographic, Clinical, and Surgical Variables

Demographic variables included sex, body mass index (BMI), age, tumor height, American Society of Anestesiologist (ASA) score, and Magnetic Resonance Imaging (MRI)-based tumor stage. Intraoperative variables included surgical technique and approach, conversion rate, number of stapler firings, type of anastomosis, use of ICG, endoscopic grading, air leak test results, operative time, and stoma formation. Postoperative outcomes included all complications occurring within the first 90 postoperative days. Postoperative mortality was defined as death within 90 days after surgery or before hospital discharge. Complications were classified according to the Clavien–Dindo system as minor (Grades I–II) or major (Grades III–IV). Patients were followed-up over a two year period.

### 2.8. Definitions

Feasibility was defined as the completion rate of each component of the protocol.

Safety was defined as the absence of adverse events (e.g., anastomotic disruption due to distension, iatrogenic injuries) following endoscopic assessment or other testing modalities.

Anastomotic leakage was defined as a defect in the intestinal wall at the site of the colorectal or coloanal anastomosis (including suture and staple lines of neorectal reservoirs) resulting in communication between the intra- and extraluminal compartments. A pelvic abscess adjacent to the anastomosis was also considered an anastomotic leak [23].

Endoscopic mucosal grading to assess anastomotic integrity in all patients was performed according to the Sujatha-Bhaskar et al. [21]:

- Grade 1: Circumferential normal-appearing peri-anastomotic mucosa-
- Grade 2: Mucosal ischemia/congestion involving <30% of either colonic mucosa or rectal mucosa
- Grade 3: Mucosal ischemia/congestion involving ≥30% of either colonic mucosa or rectal mucosa or ischemia/congestion involving both sides of the staple line.

### 2.9. Ethical Considerations

This study was conducted in accordance with the Declaration of Helsinki and Good Clinical Practice guidelines. The hospital’s ethics committee approved the study (No. 2022.045). All patients were informed prior to surgery, and written informed consent was obtained before the intervention. Once the surgeon confirmed intraoperatively that the patient met the inclusion criteria, the protocol was applied. Patients retained the right to withdraw from the study at any point and were excluded if any exclusion criteria emerged during follow-up.

### 2.10. Statistical Analysis

Descriptive analyses were performed for demographic, clinical, surgical, and postoperative variables, as well as CASPI protocol assessments. Continuous data were expressed as mean ± standard deviation (SD) when normally distributed or as median and interquartile range (IQR) otherwise. Student’s *t*-test was used for normally distributed continuous variables, and the Mann–Whitney U test for non-normally distributed variables.

Categorical data were expressed as counts and percentages and analyzed using Fisher’s exact test or χ^2^ test. Statistical analyses were performed using IBM SPSS (version 24), with significance set at *p* < 0.05.

## 3. Results

### 3.1. Patient Characteristics

**Figure 2:**
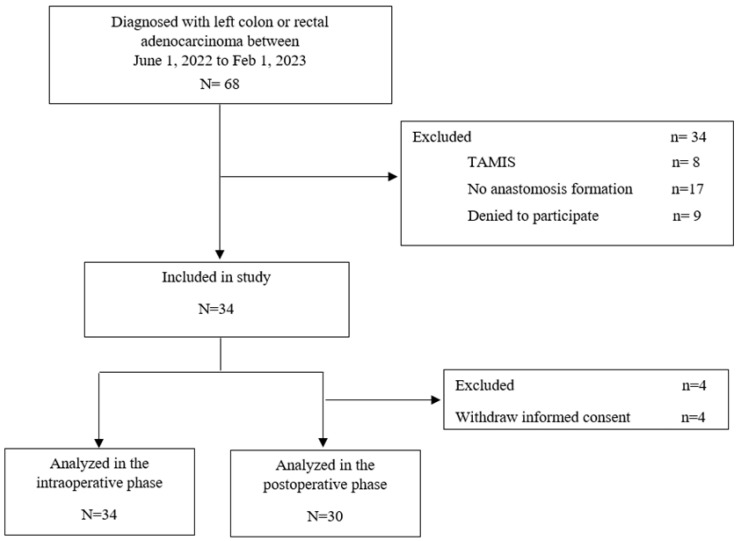
Flowchart of the study.

Among the 34 included patients, 12 were female (35.3%) and 22 were male (64.7%) (Figure 2). The median age was 63.5 years (IQR, 56–69.8), and the median BMI was 27.7 kg/m^2^ (IQR, 24.5–29.9). A total of 31 patients (91.2%) were classified as ASA, with the remaining 8.8% (3 patients) classified as ASA II (Table 1).

Thirteen patients were diagnosed with colorectal tumors located less than 10 cm from the anal verge, and 21 patients had tumors located >10 cm from the anal verge. Among patients staged by MRI (86.7%), 2 patients were staged as cT1 (7.1%), 8 as cT2 (28.6%), 15 as cT3 (53.6%), and 3 patients as cT4 (10.7%). Five patients (15.2%) had synchronous metastases at diagnosis. Of the 27 patients with rectal tumors, 18 patients (66.7%) received neoadjuvant treatment (Table 1).

### 3.2. Surgical Characteristics and Postoperative Outcomes

The most frequent surgical technique was anterior rectal resection (18 interventions, 52.9%), followed by low rectal resection (11 interventions, 32.4%), sigmoidectomy (4 interventions, 11.7%), and one total colectomy (2.9%) (Table 2).

The surgical approach used was robotic in 25 interventions (73.5%), seven surgeries performed trough laparoscopy (20.6%) and two trough TaTME (5.9%). No conversion to open surgery was required. End-to-end anastomosis was performed in 32 patients (94.1%), and end-to-side anastomosis was performed in two patients (5.9%).

Three types of circular staplers were used for the anastomosis. A 29 mm stapler was used in 27 anastomosis (79.4%), a 31 mm stapler in 17.6% (6 anastomosis), and a 33 mm stapler in one patient (2.9%). Thirteen anastomoses were performed at <5 cm from the anal verge.

Temporary protective ileostomies were performed in 12 patients (35.3%). The median surgical duration was 210 min (IQR, 165–280).

Four patients experienced postoperative complications. Two patients were diagnosed with paralytic ileus, one had phlebitis, and the other had a pneumothorax that was resolved with chest drainage. None of the 34 patients required re-intervention; however, one patient was readmitted due to dehydration from the ileostomy (Table 2). The median hospital stay was 5 days (IQR, 4–6).

Anastomotic integrity was observed intraoperatively and postoperatively in all patients who underwent endoscopy.

No early or late anastomotic leakage or other complications associated with colorectal anastomosis were observed within two years after surgery. No reinterventions or readmissions related to anastomotic complications were registered. Stoma closure was performed in all patients with temporary ileostomy.

### 3.3. Adherence of the CASPI Protocol

All 34 patients included in the study completed all the intraoperative components of the CASPI protocol (Table 3).

Using ICG, adequate perfusion at the anastomotic site was observed in 32 (94.1%) patients. In two cases, the predefined transection point was based on the perfusion findings. No cases of anastomotic ischemia were registered.

A total of 68 intact doughnuts were obtained (100%), with diameters and heights calculated in 94.1% of the cases. In the proximal doughnut, the median diameter was 18 mm (IQR, 16–20), and the height was 10 mm (IQR, 9–12). In the distal doughnut, the median diameter was 17 mm (IQR, 15–20), and the height was 5 mm (IQR, 5–8).

An air leak test was also conducted in 100% of the patients, and no positive results were observed.

**Figure 3:**
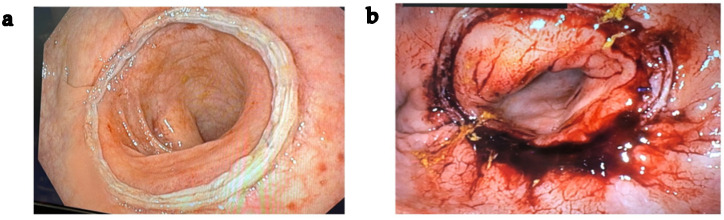
Patterns of IOE founded in our pilot study. (**a**) Grade 1: Circumferential normal-appearing peri-anastomotic mucosa. (**b**) Grade 2: Mucosal ischemia/congestion involving <30% of either colonic mucosa or rectal mucosa.

According to the intraoperative endoscopy grading system, the majority of patients (26/34, 76.5%) exhibited Grade 1 anastomotic mucosa, indicating normal-appearing peri-anastomotic tissue (Figure 3a). The remaining 8 patients (23.5%) were classified as Grade 2, showing localized mucosal ischemia or congestion affecting less than 30% of either colonic or rectal mucosa (Figure 3b). No cases of Grade 3 mucosa, were observed.

Regarding additional morphologies observed in intraoperative endoscopy images, nine patients presented with “dog ear” structures, and one of these patients underwent anastomotic reinforcement using sutures.

The compliance rate of postoperative endoscopy was 88.2% (30 patients) (Table 3). Three patients declined the second endoscopy, and one patient was unable to attend because of readmission.

### 3.4. Safety of the CASPI Protocol

None of the 34 patients experienced complications associated with the intraoperative and postoperative tests performed using the CASPI protocol. There were no iatrogenic injuries, anastomotic disruptions, or complications associated with endoscopy, ICG administration, or leak testing.

## 4. Discussion

This single-arm pilot study demonstrated high adherence to the CASPI protocol, with 100% compliance for intraoperative steps and 88.2% for postoperative assessments. Each component of the protocol was proven feasible and safe after implementation. In terms of postoperative assessment the adherence was lower underscores logistical and patient-related challenges in follow-up sigmoidoscopy. Both intraoperative and postoperative endoscopic evaluations of the colorectal anastomosis revealed no adverse events.

Previous studies by Emile et al. [24] described the use of a quadruple assessment of colorectal anastomoses. Tirelli et al. [25] investigated the combined use of perfusion and technical tests intraoperatively to reduce postoperative complications in TaTME, while Kryzauskas et al. [26] proposed a comprehensive trimodal assessment and concluded that their approach enabled the identification of early technical failure. The CASPI protocol was developed with the aim of standardizing colorectal anastomosis assessment using a multimodal approach.

The use of ICG imaging in colorectal surgery allows assessment of perfusion, guides intraoperative adjustments, and has been associated with reduced leak rates and improved patient outcomes [27,28,29,30,31]. In our pilot study, all patients underwent ICG testing, with photographic documentation performed intraoperatively; however, adequate perfusion was obtained in the 94.1% of the patients.

ICG perfusion assessment is qualitative and operator-dependent, and interobserver variability was not formally assessed in the present study. Emerging quantitative methods, such as maximum intensity, relative maximum intensity, time-to-maximum intensity, and in-flow parameters for ICG [32,33], may reduce subjectivity and improve reproducibility in future studies.

The integration of ICG imaging and intraoperative endoscopic mucosal evaluation has been proposed by Kryzauskas et al. [34] and Vallicelli et al. [35] for assessing colorectal anastomoses. However, further studies are needed to determine the added value of combining ICG angiography with intraoperative endoscopy (IOE).

Several studies have shown reductions in anastomotic complications with the use of intraoperative flexible endoscopy [19,21,35,36,37]. This technique allows direct assessment of mucosal alignment and detection of bleeding or defects at the anastomotic site [38]. Although endoscopic surveillance of colorectal anastomoses has historically raised concerns due to the presumed risks of insufflation, available evidence does not support these concerns. The maximal pressure generated during IOE in humans is approximately 42 mmHg, whereas at least double this pressure is required to cause leakage in large-animal models [26,39,40].

The incorporation of intraoperative endoscopy for anastomotic assessment complements standard evaluations (ICG perfusion, donuts integrity, and air leak test) by providing direct visualization of the mucosal surface and anastomotic line, allowing detection of subtle mucosal irregularities or points of tension. The identification of “dog ear” structures in nine patients, with one requiring suture reinforcement, illustrates the added value of intraoperative endoscopy in detecting subtle technical irregularities that may not be apparent from perfusion or doughnut assessment alone.

The use of intraoperative endoscopy also allows mucosal grading, which may further guide intraoperative decision-making and improve patient outcomes [21]. In our study, two mucosal patterns were identified, differing from the three grades described by Sujatha-Bhaskar et al. [21]. As mentioned above, interobserver variability exists in grading anastomotic mucosa, which may influence the subsequent management of patients classified as Grade 2 or 3. Patients classified as Grade 2 mucosa intraoperatively were monitored more closely postoperatively trough polymerase chain reaction measurements and imaging. Therefore, it is important to consider these limitations when interpreting intraoperative mucosal assessments and to standardize training and protocols to enhance consistency.

Unlike the findings of Kryzauskas et al. [26], who reported high leak rates despite technically well-performed anastomoses, our series had no early anastomotic leaks following intraoperative assessments. However, it is important to note that this was a single-arm pilot study without a control group; therefore, no causal inference can be drawn regarding the effect of the CASPI protocol on anastomotic outcomes. The absence of anastomotic leaks in our cohort should be interpreted as a descriptive finding rather than as evidence of clinical efficacy.

Apart from the intraoperative assessments, the CASPI protocol incorporates a second postoperative endoscopy (flexible sigmoidoscopy), which provides objective confirmation of anastomotic healing. This is particularly relevant for patients scheduled for adjuvant therapy or for those with temporary stomas prior to closure. All anastomoses assessed postoperatively showed no defects. The implementation of postoperative endoscopy in clinical practice may help detect late-onset leaks more accurately. Ikeda et al. [40] also demonstrated the safety and clinical value of endoscopic evaluation in suspected anastomotic leaks, particularly for assessing technical factors, and reported a reduced need for defunctioning ileostomy.

The strengths of this study include the high adherence rate achieved by a dedicated surgical team and systematic visual documentation, which may have contributed to protocol compliance. Our findings align with those of Guyton et al. [41], confirming that such protocols are feasible and useful for serially monitoring anastomotic healing.

This study has several limitations. It was a single-center pilot study with a small sample size, and heterogeneous population, which restricts generalizability and statistical power. The absence of anastomotic leakage should not be interpreted as evidence of the protocol’s efficacy. Adherence to postoperative sigmoidoscopy, although high, was slightly lower, reflecting logistical and patient-related challenges. Additionally, ICG perfusion assessment and intraoperative mucosal grading are qualitative and operator-dependent. Finally, several factors, including high surgical expertise, the predominance of minimally invasive procedures, and patient selection, may have contributed to the observed outcomes independently of the protocol. Future studies should include an appropriate control group to properly assess the clinical impact of the protocol and to disentangle its effect from other contributing factors.

## 5. Conclusions

The CASPI protocol demonstrates feasibility and safety for the standardized assessment of colorectal anastomoses. In our series, no adverse events were reported. Further studies are needed to validate these findings and support the broader implementation of the CASPI protocol in clinical practice.

## Figures and Tables

**Table 1:** Patients demographic clinical and surgical characteristics.

	N = 34
Sex	
Male	22 (64.7%)
Female	12 (35.3%)
Age [median (IQR)], years	63.5 (56–69.8)
BMI [median (IQR)], kg/m^2^	27.7 (24.5–29.9)
ASA score (%)	
II	31 (91.2%)
III	3 (8.8%)
Tumor location	
Left colon-sigmoid	7 (20.6%)
Rectum	27 (79.4%)
cT	
T1	2 (7.1%)
T2	8 (28.6%)
T3	15 (53.6%)
T4	3 (10.7%)
Synchronous metastases	5 (15.2%)
Neoadjuvant treatment	18 (66.7%)
Surgical Technique	
High anterior resection	18 (52.9%)
Low anterior resection	11 (32.4%)
Sigmoidectomy	4 (11.7%)
Total colectomy	1 (2.9%)
Approach	
Robot	25 (73.5%)
Laparoscopy	7 (20.6%)
TaTME	2 (5.9%)
Circular staplers	
29 mm	27 (79.4%)
31 mm	6 (17.7%)
33 mm	1 (2.9%)
Anastomosis	
End-to-end	32 (94.1%)
End-to-side	2 (5.9%)
Conversion	0 (0%)
Defunctioning ileostomy formation	12 (35.3%)
Operative time (min) [median (IQR)]	210 (165–280)

BMI, body mass index; ASA, American Society of Anesthesiologists; IQR, Interquartile range.

**Table 2:** Postoperative variables.

	N = 34
Overall complication	4 (11.7%)
Clavien–Dindo classification	
II	3 (75.0%)
IIIa	1 (25.0%)
Re-admission	1 (2.9%)
Re-intervention	0 (0%)
Paralytic Ileus	2 (5.9%)
Early anastomotic leakage	0 (0%)
Late anastomotic leakage	0 (0%)
Hospital stay (days) [median (IQR)]	5 (4–6)

IQR, Interquartile range.

**Table 3:** Adherence to CASPI protocol.

	N = 34
Indocyanine green	34 (100%)
Doughnuts integrity	68 (100%)
Air leak test	34 (100%)
Intraoperative Endoscopic	34 (100%)
Postoperative endoscopy	30 (88.2%)

## Data Availability

Summarized anonymised data will be made available on request.

## Abbreviations

The following abbreviations are used in this manuscript:

AL	Anastomotic leakage
ALT	Air leak test
CASPI	Colorectal Anastomosis Safety Protocol Implementation
ICG	Indocyanine green
DI	Doughnuts integrity
IOE	Intraoperative Endoscopic
PE	Postoperative endoscopy
MRI	Magnetic resonance imaging
ASA	American Society of Anesthesiologists
BMI	Body mass index
IQR	Interquartile range
SD	Standard deviation
